# Promoting Construction Industrialisation with Policy Interventions: A Holistic Review of Published Policy Literature

**DOI:** 10.3390/ijerph182312619

**Published:** 2021-11-30

**Authors:** Xin Jin, Geoffrey Q. P. Shen, Qian-Cheng Wang, E. M. A. C. Ekanayake, Siqi Fan

**Affiliations:** 1 Department of Building and Real Estate, The Hong Kong Polytechnic University, Hung Hom, Kowloon, Hong Kong, China; verna.x.jin@connect.polyu.hk (X.J.); geoffrey.shen@polyu.edu.hk (G.Q.P.S.); anushika.ce.ekanayakemudiyanselage@connect.polyu.hk (E.M.A.C.E.); 2 Department of Land Economy, University of Cambridge, Cambridge CB3 9EP, UK; qw250@cam.ac.uk

**Keywords:** construction industrialisation (CI), policy intervention, sustainable urban development, classification analysis

## Abstract

By adopting the concept of ‘factory assembly followed by onsite installation,’ construction industrialisation (CI) plays an increasingly important role in sustainable urban development. CI can enhance construction quality and efficiency while reducing environmental impacts. To promote the CI, several policy interventions have been developed and implemented in different countries and regions. This study reviews the global CI promoting regulations and policies to provide a comprehensive insight into its interrelationship and development tendency. The research selects 105 publications related to practical CI policy from widely utilised databases (i.e., Web of Science and Scopus). Based on the annual publication trend analysis, geospatial distribution, and citation analysis, seven interrelated critical CI policy formulation themes are identified and examined: regulatory policies, standardised policies, promotional policies, urban design and planning policies, technological policies, managerial and educational policies, and sustainability policies. In addition, internal correlations and mutual influence among these seven classified policies are explored and discussed, which helps scholars enhance their grasp of current CI policy research and guide future research. This review provides the research community and industrial practitioners with a comprehensive understanding of various CI-promoting policies and a roadmap to CI-promoting policy development and evaluation.

## 1. Introduction

Construction industrialisation (CI), as an innovative and effective manufactory-based construction mode, has been progressively adopted worldwide over the past few decades as an alternative to the traditional onsite construction method. CI encompasses extensive use of prefabrication, which is a manufacturing process generally carried out at a specialized factory, where a variety of building materials are combined to shape a fabricated component to be installed onsite as part of the construction process [1]. Despite being not an especially new concept, industrialized building consistently attracts fresh interest and kickstarts investment boom in the wake of the improvement of technology and changes in the global economic environment [2]. Growing awareness of the significance of CI in improving construction quality and efficiency, and elevating environmental performance, has led to increased scrutiny of supportive policies and regulations by many countries and regions [3]. The advantages of CI include reduced construction time [4], better-controlled quality of construction [5], enhanced productivity and durability [6], reduced construction waste [7], less-skilled workforce required onsite [8], and improved occupational health and safety [9].

The surge of CI has ameliorated the status of the construction sector in housing crises and lagging productivity. Nowadays, the maturing of the CI has profoundly changed the mindset of the incumbents, radically reshaped the way people build, and is currently experiencing a new wave of attention and investment. Even anchored in diverse construction surroundings and social backgrounds, many countries pragmatically impose promotional policies of CI with a relentless focus on mandatory requirements and incentive schemes. The need for comprehensive CI policies, including the sustainable development debate over different contexts, are now at the centre of social and economic dialogue. Meanwhile, the applications of CI are widely seen in Hong Kong (HK) [10], Singapore [11], Mainland China [5], Malaysia [12], and Japan [13], and is currently in high demand [14].

The constellation of market and government policies with substantial CI stimulus plans is generating invaluable opportunities for the widespread use of advanced industrialized methods in the construction industry. Therefore, systematic research of classification and integration of previous studies on CI policy and analysis can considerably contribute to the government’s comprehensive understanding of the impacts of different policies on the CI implementation, unlocking the realization of policy targets and inspiring policymakers. Although there are many existing literatures discussing the characteristics and applications of CI, few studies have summarized and probed the interplay and confluence among the categorized policies on a global scale. This research aims to present a holistic knowledge of the current status of the published articles relevant to CI policies. Some fundamental data analyses of the involved papers are shown, such as annual publications trends, geospatial distribution, and citation statistics; seven themes of CI policy formulation from relevant studies are identified, and internal correlations and mutual influence among policies are explored and discussed.

## 2. Background of CI-Related Policies

Many countries are committed to ensuring the durability of their entire housing stock through extensive use of prefabricated components and precast elements, which is reflected by the percentage of prefabricated volume and the variety of prefabricated assembly units [15]. Scaling up the industrialized construction would effectively satisfy the affordable housing supply and meet the climbing demand, especially in metropolitan cities like HK [16]. Considering the superior performance of CI in housing developments, although, under different construction environments and different social conditions, various regions have released relevant policies on compulsory regulations and incentive schemes to promote CI uptake [17].

Since HK is one of the most densely populated megacities in the world, housing supply is a crucial factor in its sustainable development [18]. With the overwhelming demand for high-quality, affordable housing in HK over recent years, the HK government has implemented compulsory regulations and incentive schemes to adopt CI [17]. As for manifestations of CI, prefabricated concrete components (PCCs), were first applied in public housing developments in the 1960s, where a 16-story public housing block was constructed with precast concrete panels [19]. Since the mid-1980s, the Hong Kong Housing Authority (HKHA), the main provider of public housing in HK, has mandated the use of prefabricated units, together with standard modular integrated design, for all public housing projects [20], which greatly enhanced the development of CI applications in HK. On the other hand, the HKHA has been promoting CI in order to provide a more effective platform to enhance construction productivity [21]. Commonly used PCCs by HKHA involve precast concrete panels, precast façades, precast staircases, panel wall partitions, and semi-precast slabs, which would be incorporated into subsequent public housing development projects together with precast concrete technology [22]. From 2001, the HK government decided to provide Gross Floor Area (GFA) exemption for the building projects adopting CI and green technologies, which was reflected in the Joint Practice Note 1 and 2 issued by the Hong Kong Buildings Department (HKBD) [23,24]. These incentive schemes enable real estate developers to access additional floor areas [25] with expenditure savings of up to 4–6% [26], which motivates private developers to apply CI. The greater use of CI was further promoted in the Chief Executive’s 2017 Policy Address [27], which implemented new technologies to improve productivity and cost-effectiveness, such as CI, to build large-scale construction projects to improve the level of construction automation. This policy has benefited small and medium scale enterprises [27]. Since then, more unique and efficient design has been introduced through prefabricated metal railing design with the single aspect block design [28]. For example, prefabricated components are widely used in the construction of infrastructures such as the immersion tunnel, the HK-Zhuhai-Macao Bridge, and the HK-Shenzhen Corridor.

In Mainland China, more than 2 billion square meters of building area have been built in recent years. A study conducted in the scale of China has shown that supportive policies can significantly escalate labour productivity, meanwhile saving the construction materials [29]. Nevertheless, CI has not been totally accepted in Mainland China even though the area of new buildings is more than half of the world’s total. A study has shown that less than one per cent of new construction in China is built by modular integrated construction (MiC) [30]. The report shows that China’s construction market will increase over the next 20 to 30 years with the total amount of new construction area reaching 20–30 billion square meters [31]. The Ministry of Housing and Urban Rural Development (MHURD) proposed that more than half of construction shall adopt MiC by 2020. CI is considered valuable in promoting sustainable practices in China, wherein large-scale urbanization plans have been established [32]. The use of CI has been incorporated into the 12th Five-Year Plan [33] and the 13th & 14th Five-Year Plan. Many cities also provide regulations for compulsory use or incentive schemes toward CI uptake based on their specific conditions [34]. For example, in Beijing, Shanghai, Shanxi and Wuhan, projects that adopt CI can obtain floor area incentives for economic benefits; in Hefei, CI projects pay fewer initial fees to the government; in Shenzhen, all public projects must incorporate CI in their buildings; in Shenyang, the use of CI should be included in tendering contracts for particular projects. Overall, China has gradually developed into a diversified and comprehensive policy portfolio encompassing initiatives, guidance, standards, regulations and incentives, shifting from the initial gestation stage [14,35].

Japan possesses the most valuable experience in developing CI and a remarkable market share. In the 1970s, Japan established a subsidy system to promote CI uptake in residential buildings. This subsidy system was followed by exploitation grants for developing manufacturing technologies for residential systems. The government provides loans with 30% lower interest rates than commercial banks for private sector CI developments [13].

Malaysia is also making efforts to facilitate CI uptake by launching the Industrialized building systems (IBS) Roadmap 2003–2010 [36] and IBS Roadmap 2011–2015 [37] for the purpose of guiding incentives, research and development programs, and other monitoring and management regulations. In 2004, all public construction projects were strongly encouraged to have at least 50% of IBS elements, assessed by the IBS Score Manual [38]. In 2006, a tax incentive was further provided through the Acceleration Capital Allowance to purchase steel moulds for precast components. In 2008, the compulsory requirements were more rigorous than those in 2004; specifically, the IBS content of projects increased to no less than 70%, and IBS implementation was included in tendering documents.

The promotion policies of CI uptake in Singapore are similar to that of HK [11]. Since 1981, Singapore’s Housing Development Board has provided interest-free loans to contractors and CI plants to support the development of CI [11]. Compulsory use of CI is enforced indirectly by statutorily complying with the buildability regulations [10]. Building designs are assessed under the Buildable Design Appraisal System and must meet the minimum requirement of a buildability score. This regulation has largely facilitated CI uptake in the private sector. Since the implementation of the regulation, the buildability score has been the significant criterion in awarding public housing projects, which provide accommodation for approximately 80% of Singapore’s population.

The relevant policies for CI and prefabrication applications are varied and cluttered, requiring a systematic review of existing literature within the scope of this study. This review can broadly provide the government and CI practitioners with a comprehensive understanding of the impact of different policies on CI implementation. It will inspire decision-makers in examining the realization of policy targets.

## 3. Research Methodology

### 3.1. Data Collection

A systematic literature review (SLR) methodology is adopted in this study. This review method [4,15,39] has provided guidance on picking out target academic articles that fit the topic in CI research. Accordingly, systematic analysis of published papers is widely used for reviewing and exploring various knowledge domains [40]. Given its advantages such as efficiency, availability, stability and cost-effectiveness [41], a systematic review of literature is applied in this study.

The authors selected the Web of Science (WoS) core collection and Scopus as the databases to search for articles applicable in this study since they are the most frequently used international databases for conducting literature reviews [42]. Considering that this study focuses on Construction Industrialisation Policies (CIP) research domain, combinations of the keywords are set. Namely, ‘construction industrialisation’, ‘industrialized building/housing’, ‘modular construction/building’, ‘prefabrication,’ ‘prefabricated construction/building,’ ‘precast concrete’, ‘offsite construction’ and ‘policy*’ (where * means policy or policies in searching) are searching criterion. Documents consisting of these phrases in their title or abstract or keywords were examined in the study. The purpose is to acquire the original and review papers on CIP, making the results more accurate and convincing. This study manually eliminated papers and conference proceedings that are less relevant to the topic. Therefore, the search results were purified by refining the document types as articles or reviews, and based on the science categories of environmental sciences, engineering environmental, green sustainable science technology, engineering civil, construction building technology, engineering industrial and urban studies. Nonetheless, some irrelevant papers still appeared in the search results under the rigorous selection criteria. After a thorough scanning of the abstracts of these articles, the unrelated papers were excepted. Eventually, 105 CIP-related papers were filtered and retrieved for further review, including 99 research articles and six review papers.

### 3.2. Data Analysis

#### 3.2.1. Annual Publications Trend on CIP Related Research

**Figure 1:**
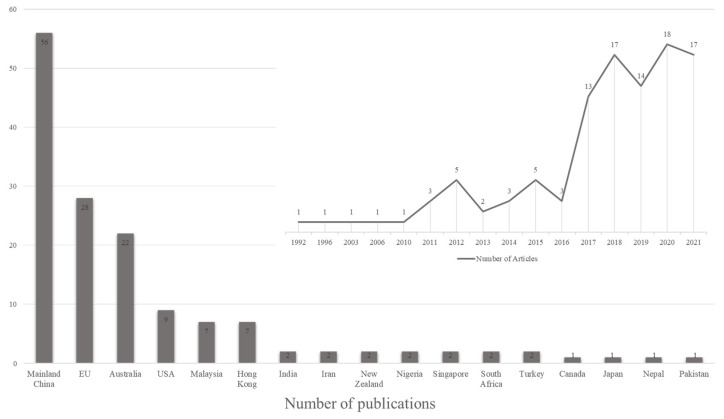
Annual publications trend and Geospatial distribution of CIP articles.

Based on the systematic and comprehensive search in the WoS and Scopus databases, 105 publications related to CIP over the period to 2021 were extracted and analysed further in this study. The development of the knowledge domain spanning between 1992 and 2021 is illustrated in Figure 1, which indicates the annual publications trend of CIP.

Figure 1 shows the variations of the selected yearly journal publications on CIP during the 30-year research period (1992–2021). The previous 30 years can be further subdivided into three stages: (1) 1992–2010 when the emergence of CIP maintained a relatively slow pace of development with only five papers recorded during this period; (2) 2011–2016 when the emergence of this topic had been on the rise, varying between 2 to 5 yearly; and (3) since 2017 the annual academic papers has risen steeply to 13 or more, with the highest number of 18 in the year 2020. It is worth noting that the publications in 2021 are incomplete as the chosen papers were up to July of 2021. Figure 1 also highlights that the CIP research area gained a rising interest among researchers over the last decade. This is validated by the findings of Li et al. [15] that CI is becoming progressively valuable to the whole construction manufacturing, along with the research topic named guideline and policy in CI. The CIP had been identified as the principal research direction of industrialized building studies following the development of some innovative technologies and approaches. Therefore, under this current increasing trend, it is expected that the CIP implementation would keep enriching over the intervening years.

#### 3.2.2. Geospatial Distribution of the Involved Papers on CIP Related Research

Academic and public studies in different regions are more targeted and provide significant guidance to the formulation of local policies in corresponding industries [43], particularly in the construction industry. Wuni et al. [44] also illustrated that the construction field in one country, where previous research practise is not available, could learn experiences from other nations to design policies in line with their own circumstances. Thus, it is of significance to emphasize the geographical distribution of the involved publications on CIP for promoting CI application. The included literature is categorized by their geographical context, as indicated in Figure 1, showing geospatial distribution.

The statistical records in Figure 1 reveal that 17 different regions have contributed to the CIP implementation research. The sample provides a typically broad perspective on CIP promotion within the globe, for developing, transition and developed economies are all included. Some countries have more leverage than others in the CIP research discourse. The slightest territorial contributors contain Canada, Japan, Nepal, and Pakistan with only one publication respectively, whereas the six leading contributing countries or regions include the Mainland China (56), European Union (EU) (28), Australia (22), USA (9), Malaysia (7) and Hong Kong (7). Among these 17 regions, China is the dominant contributor, with 56 articles in the mainland and seven publications from Hong Kong. This finding is consistent with the current situation as China is the top market for construction around the world [45] and continuously devotes many efforts to the effective promotion and implementation of industrialized residential building (IRB) policies [46]. Thus, sorting and generalizing policies in CI from these countries could suggest the advanced international experience, which could be valuable to the guidance of CI policies’ promotion.

#### 3.2.3. Citation Analysis toward the CIP Related Research

The authors of the targeted papers are recommended to indicate their reference foundation when a fresh derivative conception is proposed. In support of the findings, convincing citations should be presented as the evidence [15]. Therefore, a detailed citation index analysis, normally applied as one of the effective methods for assessing the impact of a specific journal or article, is carried out in this study. In general, a larger quantity of citations a paper records usually illustrates that this article is regarded as a pioneering publication [44]. As of July 2021, citation statistics of the involved 105 documents were examined to extract the landmark publications.

Table 1 presents the most frequently cited journals. By setting the lowest number of citations of a periodical to 40, the writers found that 11 of 50 research journals crossed the threshold. The influence of a journal in the field of CIP is assessed by its total cited counts, number of related papers published and citations per paper. Among the 11 high-referred journals, the Journal of Cleaner Production (JCR) was the most frequently cited journal, with a maximum number of citations up to 534 times, followed by Energy and Buildings (EB) with 219 referrals and Habitat International (HI) with 193 referrals. In terms of average per item, papers in HI were most cited (96.50 times per article), while those in the Journal of Management in Engineering (JME) and EB had been cited 92 and 73 times, respectively. In Table 2, the top 15 most-cited influential research papers in CIP based on citations and the sum of frequencies each article was cited is recorded. The article by Mao et al. [47] from Chongqing University was identified as the most frequently referred literature, reaching a maximum of 186 times citations totally, followed by Mao et al. [5], Cao et al. [48] and Zhang et al. [49] of 151, 104 and 80 times, respectively. The 15 pieces of literature listed in Table 2 represent the most referenced publications related to CIP. This exercise exposes an emphasis on the obstacles and challenges of CIP-related research in the context of government policy on CI, as well as comparative studies of sustainable buildings between offsite prefabrication and traditional construction methods. By implication, the successful implementation of CIP can be linked to overcoming barriers, sustainable consumption, and zero waste in CI development.

## 4. Findings and Discussion

To enhance the comprehensive understanding of CIP, the selected scientific papers were explored and categorized by conducting two steps. First, the objective policies and specific strategies in relation to CI, which were mentioned in the journal papers, were completely and carefully extracted based on their statement. Second, the categories of the CI policies within the studied period were established upon analysing and sorting the data collated in the previous step. Further, exploratory research was carried out on the discovery of internal correlations among the classified policies. The findings from this classification and interrelationships among CI policies can reveal the focus of contemporary policy for this discourse, as well as better promote the implementation of CI policy step by step.

CIP-related research has witnessed continuous growth throughout the past decades. The increase of incentives can speed up the achievements of CI in practice. The CI policy research field is characterized by its different policy preferences and directivity, from promoting the technological development of the industry to achieving overall green and sustainable industrialisation. Through a detailed and comprehensive exploration analysis and repetitive assessment, this paper identifies seven themes of formulation orientation in CI policies which concluded from relevant studies, as follows: (1) regulatory policies; (2) standardized policies; (3) promotional policies; (4) urban design and planning policies; (5) technological policies; (6) managerial and educational policies; and (7) sustainability policies.

Among these seven CI policy types, regulatory policies refer to the regulations and policies formulated by local governments to impose controls and restrictions on the implementation of CI; standardized policies aim to normalize the practices of CI and help codify the best practices and technical requirements; promotional policies are the ones carried out by governments to stimulate the markets, usually pursued by various stimulus initiatives and incentive measures; urban design and planning policies tackle the issues while bringing the CI into urban design and urban development; technological policies focus on the application of emerging CI technologies and innovations which can be better reflected in CI practice; managerial and educational policies provide guidance on workforce management and effective educational programs to the relevant stakeholders in CI; and sustainability policies aim to help achieve sustainability around the use of CI from economic, social and environmental aspects. The classification of this study is established from the perspective of instrument purpose and mandatory degree. As defined by Mao et al. [5], several clusters associated with critical factors in CI practice included regulations, technological innovations, and economic incentives. Luo et al. [32] emphasized the governments’ leading role in promoting the standards for CI buildings and enhancing management and education practices. When analysing prefabrication policies, Gao et al. [29] revealed urbanization and sustainable development could encourage the widespread use of CI. In line with the existing groupings related to CI research, this study classified the seven policy types by relating CI to the regulative system, economic development and sustainable conditions. However, there is still a limitation: as is well known, the nature, purpose and impacts of policies are pluralistic and not absolute. Moreover, the criteria for this categorization are not absolute. Therefore, the categories are not utterly separate from each other. Such as the ‘housing policy’ pertains to ‘regulatory policies’, while it is also slightly related to ‘urban design and planning policies’. Because when considering the degree of strictness by government, ‘housing policy’ is an obligatory instrument and be treated as the cornerstone of other policies. The following sections describe the structured framework of these specific topics in detail.

### 4.1. Regulatory Policies

Literature on the first category mainly focuses on the policies and regulations formulated by the governments to impose controls and restrictions on CI, which consists of 11 representative policies as follows. These various policies in different countries reflect the highest degree of mandatory governance in the CI area, which have a strong implementation effect and can fundamentally affect the development of CI. The mechanisms of action for each chosen policy and its implications are explained and specified in detail.

- Public Procurement Law
- Governmental mandatory policies and regulations
- Intervention strategies
- Buildability score regulation
- Requisition–Compensation Balance policy
- EU construction policy
- Industrialized Residential Building (IRB) policy
- Advance industrialisation of the construction industry policy
- Improved real estate policies
- Housing policy
- Land Development Plan & Control & Supply & Restructure Regulation

The Public Procurement Law in Turkey, which has been modified extensively in recent years (2002–2014), has limited the powers of the Public Procurement Authority to adjust the public land sales and other building-based government procurements after the revisions, at the same time, these reforms impeded the transparency of the procurements in the real estate development sector [60], thus hindering the development of CI. The absence of governmental mandatory policies and regulations is identified as one of the top three barriers in China for CI development [5]. Han and Wang [45] also take governmental regulations into deeper consideration as one of the six important aspects to limit CI improvement by using grey DEMATEL analysis. Jiang et al. [51] emphasized the importance of governmental mandatory policies and regulations in CI development on the basis of a thorough review of 85 governmental documents and SWOT analysis. On the other hand, governmental mandatory policies and regulations are listed as the most critical driving force to transfer traditional construction to CI [61,62] and promote CI-related technology such as building information modelling (BIM) [58].

Based on fuzzy cognitive maps, Gan et al. [63] concluded that strengthening intervention strategies yields the strongest whole effect in promoting CI. The construction industry grew rapidly in Turkey between 2002 to 2006, mainly supported by state interventions [60]. The Singaporean government started to enforce the buildability score regulation in 2001, in which a minimum buildability score in building designs is required, aiming to accelerate the diffusion of CI into the private sector [11]. Requisition–Compensation balance policy is helpful to narrow the spatial discrepancies between obtainable construction land and existing developing requirement [64], which is a main foundation of the CI process [65].

Regarding the policies which are directed towards any specific country, the EU construction policy was established to standardise functional building codes [66], and China introduced the IRB policy for construction enterprises that still need to improve [46,49]. Dou et al. [67] suggested that the government should impose advisable policies with a higher priority of highly-economical provinces to achieve the differentiated promotion of the prefabricated construction. The development of advanced industrialisation of the construction industry policy and improved real estate policies are proved to be an applicable way to manage the housing bubble [68].

Housing Policy, such as ‘measures of Beijing on the reward for industrialized housing (IH) residential Projects’ which was issued in 2010 [69] and other measures about introducing the CI into the current construction system in Romania [70] and the USA [71], as well as adjusted rents and the focus rearrangement from hiring to ownership in the Czech Republic [72], are dedicated to proactively encouraging the popularization of CI. The law on land development plans and control in Turkey made the procedures for acquiring building permits more simplified, thus promoting property development [60]. Actually, land supply regulation is an intermediate step, which serves for improved real estate policy [68]. Land-restructuring regulation in China indicates the ‘increasing versus decreasing balance’, which plays a major role in rural construction industrial development [73].

In developing countries, such as Libya, the development of prefabricated buildings is considered to be at a starting point, as most of the industrial practitioners were unable to receive plentiful knowledge of regulatory policy guidelines [74]. In the developed context such as the U.S., regulatory barriers are fundamentally recognized in the construction industry in order to apply circular economy principles, especially the inconsistency of regulations at city, state and federal levels are found [75]. The government shall play a leading role in promoting perfricated construction, and among the policy factors, the regulatory mechanism is revealed to be a very influential factor [65]. After a comprehensive understanding and generalization, it concluded that regulatory policies enforced by governments are a critical and indispensable cornerstone for the CI promotion to the construction industry.

### 4.2. Standardized Policies

Articles about the second category of the CI policies mainly concentrate on standardized policies, which help codify best practices and technical requirements to ensure structures are safe and proper. As shown below, the standardized policies contain six specific policies. Policies classified into this category also represent a high degree of mandatory governance. However, different from regulatory policies, these six policies are all about design norms and guidelines that are more specific and targeted. For each standard, a detailed description of its mechanisms and implications are presented.

- Regional precast construction standards
- Building prefabricated components design codes standards
- Design level standards
- Construction quality acceptance criteria
- Prefabrication technical and construction method standards
- Site selection criteria

According to Wang et al. [76], low standardization was listed as a major barrier for CI promotion. In mainland China, the regional level policy associated with precast construction standards is biased, Zhu et al. [77] revealed that additional emphases and efforts on precast construction standards were put on the central rapid-developing areas such as Yangtze River Delta (8 standards) and Bohai Economic Rim (5 standards). While the specific and targeted standards relevant to the CI were still rare in the ‘backward’ regions such as northwest and southwest of China (1 standard). Therefore, refining and enhancing the regional precast construction standards, especially in some less developed areas, is truly necessary and quite urgent to achieve the overall highly evolved industrialisation in China.

Additionally, the inappropriate or even absence of design codes and standards for prefabricated components in industrialized buildings is identified as a critical political factor related to inefficient adoption and poor performance of CI [5,45,49]. Both developed and developing regions had issued regulations associated with building design codes compliance, such as the updated ‘Code of Practice for Precast Concrete Construction 2016′ announced by the HKBD [78]; the ‘precast concrete construction handbook’ prepared by the Hong Kong Institution of Engineers [79]; the China State Council announcement [80]; 76 local codes for assembly building design issued by 24 provinces and autonomous regions [51] in Mainland China; EU policies about creation and harmony of building codes [66] in EU; and improvement of design guide and new codes and standards is proceeding in New Zealand [81] and Australia [82].

The relatively low level of standardization in design was recognized as one of the major barriers and challenges by Li et al. [83] in the piping prefabrication areas. After a thorough literature review and an in-depth interview with CI industry professionals, Han and Wang [45] added the lack of construction quality acceptance criteria as an identified obstacle to CI adoption. In reality, the reliance on conventional construction methods is still one of the foremost three barriers to CI adoption [5]. Thus, enhancing prefabrication technical standards and optimization of construction methods [82] is urgently essential in the emerging CI market [58]. A range of housing technology standards, listed as ‘Technical Assessment for Residential Projects’ and ‘Standards for Modular Combination of Residential Houses’, and so on, have been supported by the Chinese authority for the CI development [69]. The site selection criteria are also worth exploring, which contributes to the CI development because the location of prefabricated plants has been a concern. A study was conducted by Azman et al. [84] on the site selection criteria after the endorsement of CI application in domestic buildings in Malaysia, in which 15 site selection criteria were analysed to obtain the optimal one.

The standardized policies mentioned emphasized the leading role of government in its enforcement and promotion of CI to construction manufacturing, indicating the relevant standard specification system should be continuously improved over time.

### 4.3. Promotional Policies

The third category of the CI policies explores what stimulus initiatives and incentive measures have been introduced to vitalize the market and increase the share globally. The eight specific policies listed are economic incentive instruments rather than mandatory policy orientation. Various countries and regions develop their specific promotional policies from different perspectives, depending on local conditions and CI developmental stage. For each chosen policy, mechanisms and implications should slightly differ between them.

- Industrialized housing and offsite construction (OSC) adoption policies
- Design-build contracts encouraging policy
- CI market acceptance encouraging policy
- IH residential projects rewarding policy
- Governmental economic incentive push or supportive policies
- Financing supporting schemes
- Governmental preferential policies
- Government subsidies policies and welfare measures

The findings concluded by Zhang and Skitmore [69] and Gan et al. [52] indicated that formulating policies and strategies are required to encourage industrialized housing and effectively facilitate OSC adoption in China. Lu et al. [59] clarified that unnecessarily misconception about the level of prefabrication—‘the higher, the better’—instead, they developed a systematic framework to determine the optimal prefabrication adoption level under different political backgrounds. The Singaporean government has made much effort to diffuse the application of CI to the private sector, and one way was to encourage private projects to employ design-build contracts [11]. By applying the mix system method, Zhang et al. [34] took inspiring market acceptance of CI as a measure in policy management from a Chinese firm-level perspective. China also announced many policies from a regional-level standpoint. Such as issuing the ‘Measures of Beijing on the Reward for IH Residential Projects’ in 2010, in which three per cent of all construction work should be assigned to apply IH manner [69], encouraging the rapid development of CI.

Both developed and developing countries have issued corresponding administrative and economic incentives or supportive policies, such as Turkey [60], EU [85], China [5,6], Iran [86] and Singapore [11]. However, through investigation and analysis, the imperfect of government economic incentives or support has still been identified as a foremost barrier to adopting CI [6,45]. Interestingly, reputational and financial incentive policies are found to behave the most effective for real estate enterprises to adopt industrialisation. A hybrid model of prefabricated construction has indicated that the government, the developers, and buyers can share the external benefits of the prefabrication implementation by 38%, 35% and 27%, respectively [87], which could motivate their enthusiasm in future CI practices. The latest research has pointed out that incentive policies shall focus not only on real estate enterprises but also give emphasis to consumers, manufacturers and contractors [88,89]. A series of incentive schemes were proposed to further accelerate the promotion of CI applications. For example, GFA compensation for MiC is mentioned in both the Joint Practice Notes No. 1 and No. 2, which is carried out by the HKBD [59,90,91].

Particularly regarding economic incentives, fund support, tax privileges and floor area rewards are the three ways that work excellently for promoting prefabrication in the Chinese context [92,93,94]. Financial support, such as tax deduction [51,59], applies if a project has reached several requirements in applying CI. Financing supporting schemes in the EU are essential to speed up profound reformation in the CI market [85]. Governmental preferential policies in China stimulated an increasing number of contractors to invest in prefabrication housing production (PHP) [95]. Singaporean prefabrication policy included the interest-free finance provided to the PHP contractors, plants and purchasers, leading to the successful application of CI in public housing [11]. Based on simulation results, Li et al. [54] claimed that when compared with the rise of income tax incentives, subsidies provisions for CI adoption in the construction sector would generate a higher positive impact on CI promotion and construction waste reduction, which is the successful practice of Singapore [11] and China [96]. Moreover, China has added fines as a measure to achieve its goal of CI promotion [3]. Tesla [97] and Demartino et al. [98] suggested the Indian construction policies should pay more attention to improving welfare measures of building workers for CI development.

Persistent policies and incentives were revealed as critical success factors that influence the successful implementation of CI. As mentioned above, promotional policies involve a variety of forms and measures, which could be used as a means or path to further promote the CI application in the field of construction.

### 4.4. Urban Design and Planning Policies

The selected academic papers regarding the fourth category are about urban design and planning policies, which tackle how cities grow and expand. The three strategies involved in this policy type are all aimed at urban development, and the development of CI is also promoted as an intermediate means. The mechanisms and implications of these three urban policies are introduced point by point.

- Urban design and reform policy
- Distinct urban policy
- Socialist urban policy

Lehmann [99,100] indicated that it was time to bring the CI into the urban design system, and these relevant urban design strategies were beneficial to implement zero waste. Monclús and Medina [101] tried to investigate the role of urban design in the process of CI urban growth in Europe. At the same time, urban reform policy came up as a solution to improve the matter of slow growth of urban CI in Turkey [60]. Distinct urban policy in the eastern and western cities could not be overlooked to explain their unbalanced development of CI. In eastern towns, socialist urban policy leads current housing estates towards CI buildings [101]. Notably, the capacity of construction land in China stays large since highly-developed Chinese cities are still in rapid industrialisation [102], so corresponding policies have emerged. For example, the ‘National New Urbanization Plan (2014–2020)’ (NNUP) in China is a ground-breaking urbanization scheme that has been issued to help reach national urbanization targets as well as improve the competency of the construction sector, in which CI promotion is a strategy involved in NNUP.

These urban design and planning policies take promoting the development of CI as the intermediate means of its ultimate urbanization construction. Besides, it is precisely what is needed for urbanization to drive CI-relevant policy. Therefore, the urban design and planning policy is a way to expand CI adhibition, and the wide range of CI applications is a manifestation of urbanization.

### 4.5. Technological Policies

Technological policies focus on the development and promotion of CI-based technology and management innovations in the construction industry. These policies aim to promote novel CI technologies and CI-based management methods to improve the overall performance of the construction industry. These policies are further divided into three sub-categories:

- CI-based R&D promotion
- Promoting the adoption of innovative CI technologies
- Technical guidelines and standards

The first sub-category aims to promote the innovation of the construction industry by supporting research and development (R&D) of CI-based innovations. Several studies have pointed out the critical role of policy interventions to encourage both the industry and the academic community to develop novel CI-based technologies through additional incentives [3,45,99]. For example, Cao et al. [48] advised that the governments should take specific actions to promote R&D in CI-related technologies in the prefabricated residential building (PRB) industry. Guo et al. [103] also called for additional policy support in eco-friendly CI technology R&D tasks. R&D has been listed as one of the most critical driving forces towards the adoption of CI technologies in Mao’s research [5,91]. Especially with larger construction market scales, fast-growing economies, such as mainland China and ASEAN countries, are more likely to benefit from technical promotion policies. In July 2020, 13 departments of the Chinese government jointly announced the Guiding Opinions of the Ministry of Housing and Urban-rural Development [104] and Other Departments on Promoting the Coordinated Development of Intelligent Construction and Industrialized Building [104]. The guiding opinions pointed out the importance of broadening the application range of CI-based innovations and provides additional tax preferential policies for enterprises focusing on CI innovation. The policy-driven R&D has become an important promoter of the Chinese CI market, whose scale has jumped from 40.4 billion CNY to 442.0 billion CNY in 2018.

The research community has made a significant effort in the development of novel CI technologies and high-tech supporting tools. It is important to have sufficient policy interventions to promote the adoption of these novel technologies and management systems [62]. For instance, building information modelling (BIM) is an important supporting tool in CI adoption [105], which provides precise geometry information that could be used to support the CI activities to realize the prefabricated buildings [58]. Jin et al. [58] suggested that policies on BIM practice and applications also play a critical role in CI adoption. The Chinese government has formulated a series of policies to promote the adoption of BIM and its combination with CI. The Chinese government announced the Construction Industry Information Development Outline 2011–2015 in May 2011 [106] and an updated plan, the Construction Industry Information Development Outline 2016–2020 in 2016 [104]. Based on them, the Chinese government formulated a series of subsidies to support the combination of BIM and CI, while restricting contractors who use traditional methods in large-scale projects and megaprojects, which achieve remarkable results. Moreover, there are other emerging technologies, such as virtual platforms that enable visualized and automated functions throughout the life cycle of structures [107] and additive construction that extends the performances of traditional CI technologies [108,109].

In addition, IBS is another innovation that concentrates on offsite prefabrication and modularization [59,84], which presented advanced project performance in terms of cost, schedule, quality, labour demand and environmental impacts [12,32]. The research community has pointed out the important role of tailored policy interventions to promote the IBS technology adoption and implementation [110]. Based on fuzzy set theory and through a questionnaire survey conducted in China, Zhang et al. [49] reflected the perception that well-organized government supervision systems and regulatory mechanisms are insufficient at the current stage to generate enthusiasm for the CI development. To improve the operability of CIP, an exploratory study carried out by Li et al. [46] mentioned that supportive technical guidelines and instructions are essential.

Science and technology are the primary productive force, and technological policies better promote CI development through technology as an implementation approach; in turn, the broader range of CI applications promotes advances in technology. Technological advancements can help the construction industry evolve [111], which also necessitate the proliferation of technological policies.

### 4.6. Managerial & Educational Policies

Academic articles about the sixth category policies mainly concentrate on effective management and educational training among stakeholders. This policy type consists of eight individual items, and these administration strategies aim to achieve effective workforce management and are accompanied by corresponding educational programs and exercises, which are essential for the development of CI. The mechanisms of action for each selected program and its implications are clarified in detail.

- Human resource management policies
- Effective cooperation between research institutions and enterprise policies
- Proper training programs supportive policies
- Stricter safety policies
- Risk management decisions
- Preventive maintenance policies
- Company supply-chain policies
- Performance management policies

Human resource management has been identified as an important factor that could affect industrialisation policies based on existing literature [34,112]. Specifically, by applying STRO-BOSCOPE simulation techniques, Wang et al. [113] found that a reliable work scheme was more beneficial than just keeping employees busy after comparing different execution policies under variation. Resource planning has an indispensable impact on project performance after comparing different change-management policies [114]. Nasirian et al. [115] investigated different resource management policies, such as hiring multiskilled crews to optimize the prefabrication projects. Research conducted by Zhang et al. [34] illustrated that substantial exertion in policy management is needed in the local construction industry to improve its sustainable CI levels, and enhancement policies on effective cooperation among research institutions, universities and companies should be a priority.

It is found that increasing investment in designers’ professional training and strengthening relevant policies are the two efficient strategies to productively employ the prefabrication method [116]. Luo et al. [117] also implied that holding educational and training programs for all the stakeholders, including manufacturers, contractors, and clients, is a constructive way to enhance their expertise, thus reducing supply chain risks. CI requires practitioners to be equipped with higher skills and better operation with the IBS, so the government needs to provide suitable training programs effectively [32,118]. For example, the Singaporean government offered consciousness forming and skill up-gradation programs to some private sector organizations to encourage them to increase the prefabrication adoption in the educational aspect [11]. Australian researchers also claimed that skills embedded with industrialisation- and digitalization-driven technological advancements are urgently needed [119]. The attitudinal change towards CI is considered to be significant, so raising awareness with efficient education curricula in policies is necessary [99,100].

Zakaria et al. [110] suggested that stricter safety policies, such as mandatory safety awareness-building programs, should be in place to better protect the security of project staff when workers move large prefabricated components. Risk awareness is particularly important, and adopting suitable risk management policies and methods is undoubtedly necessary to promote CI application. Demartino et al. [98] had proposed a probabilistic estimation model to help stakeholders make risk management decisions when they came across the seismic risk of CI buildings. Company policy on preventive maintenance is also promising. This policy is helpful for the Just in Time implementation during CI application [120] and reduces inventory levels. The supply-chain policy of a company is distinctly critical [121]. To avoid prefabricated components production deferrals, the related manufacturers should be in reasonable control of the production line and the supply chain to manage inventories. Wong et al. [122] mentioned that construction project organizations should pay more attention to the performance management strategy when they commit to realizing institutionalized organizational change in CI promotion.

Globally, workforce management featured by collaboration, supply chain management, and educational training is a trending topic in the context of offsite construction. These managerial and educational policies promote the CI application from the perspective of organizational management and ideological education.

### 4.7. Sustainability Policies

Literature related to the seventh category of the CI policies put an emphasis on sustainability, which integrates economic, social, and environmental concerns. The nine strategies of this type listed below all revolve around the theme of driving sustainable development, with environmental protection, waste reduction and energy efficiency as branches. For each sustainability policy, a detailed depiction of its mechanisms and implications are displayed.

- Sustainable buildings supportive policies
- Environmental impacts of consumption and production reduction policies
- National environmental legislation and policy
- Green construction evaluation standards
- Construction waste reduction policies
- Extended producer responsibility (EPR) policy
- Construction and demolition wastes management (C&DWM) & recycling policies
- Carbon emissions mitigation policies
- Energy-saving policies

Nowadays, developing a circular economy to fundamentally exploit resources and step on the road to industrialisation is a frontier theme discussed by many industries [123]. Big data, supply chains, industry 4.0, carbon emission, and policy support are essential indicators of future trends in world regions [124,125]. Supportive policies of sustainable buildings are recommended as insistent measures to meet the demand and enhance the C&DWM of the construction enterprises [53]. Xie et al. [126] also emphasized that policy support of sustainability is indispensable. Lehmann [99] suggested that the government should formulate operational policies to reduce the environmental impact of consumption and production and that CI application would be a good choice. Cleaner production, together with the circular economy, plays a particularly important part in the national environmental legislation and policy, which deals with the challenges caused by environmental pollution, as well as promoting the industry’s transition towards a sustainable future.

Extended producer responsibilities can be executed by embracing green design, accepting recyclable materials and integrating low-waste technologies [127]. There are several systems or models, such as Green Construction Assessment, the Evaluation Standard for Green Construction of Buildings, and the Evaluation Standards for Green Building, which were developed in China during the last decade to evaluate the Environmental impacts (EIs) of construction projects [48]. Owing to these green construction evaluation standards, CI has been developed more quickly since PRB construction has been found to be a more effective use of energy. Construction waste reduction policies are beneficial to CI application, which needs continuous support from the government [55]. In Iran, the ‘polluter pays principle’ is treated as a practical guideline in formulating effective regulations and policies. In the UK, mass customization has been adopted as one of the main footpaths to provide a sustainable housing context [128]. In HK, more strategies have been proposed, mainly on public policies to promote waste sorting, research on the minimization and management of construction waste, development of a more mature recycling market, and government support for a green building industry [129]. Moreover, the concept of ‘zero-waste’ put forward by Lehmann [99,100] often requires higher industrialisation levels. To achieve the ‘zero-waste’ economy, the EU introduced a policy called EPR [99], which deepened the public impression of ‘zero-waste’.

The construction sector has direct impacts on the C&DWM; in order to improve its environmental performance, many regions have developed their own regulations, laws and policies, including current C&DWM regulations in China [53,68], the offsite construction waste sorting (CWS) program in HK [130,131], C&DWM regulations in Iran [86], and National Waste Strategy Policy and recycling policies in Australia [100]. CI method is proved to produce fewer carbon emissions when compared with traditional construction. Adopting a prefabricated construction method is regarded as a useful carbon emissions mitigation policy in China [34,47], encouraging a less CO_2_-intensive lifestyle [132]. Further, CI is also used as a way for carbon emissions reduction in many countries such as the UK [9], Malaysia [133], Australia [134] and New Zealand [82]. Energy-saving policies have also been adopted in China for sustainable development with the CI promotion [47].

Various stakeholders involved in the construction industry attach considerable importance to sustainability policies. Xue et al. [135] pointed out that environmental policies have noteworthy direct effects on developers to promote offsite construction, suggesting the necessity of taking the impact of sustainability policies into consideration. Jiang et al. [136] found that prefabricated manufacturers gave the highest ratings to sustainability. Liang et al. [137] and Zhang et al. [138] suggested that government decision-makers should take the actual carbon emission efficiency of the construction industry as the main basis for policy formulation and put forward effective strategies on regional sustainable development. In conclusion, sustainability policies are the most important policies for the sustainable development of mankind. The AEC industry shall work closely with the government to further achieve sustainable construction. No matter which construction methods are adopted, the goal shall be achieved in a sustainable manner.

### 4.8. Correlations among Policies

**Figure 2:**
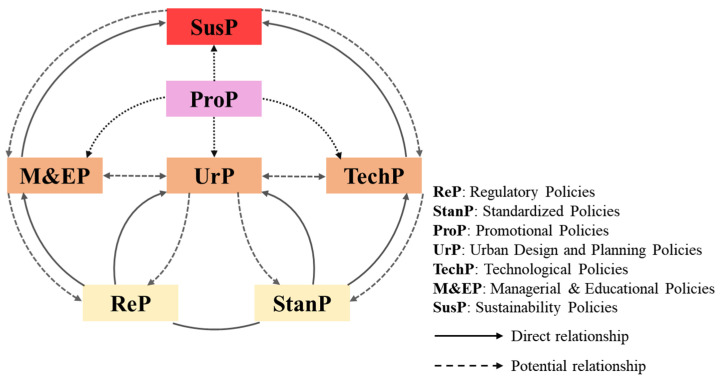
An overview of the relationships between the seven policy types.

This study sheds light on how these policies can assist and facilitate the development of CI under different contexts, comprising developing and developed countries. Strengthening policies yield the strongest overall effect in invigorating construction industrialisation [63,94]. As shown in Figure 2, the interconnections and mutual influence among these aforementioned policies are explored and discussed in detail.

Firstly, the seven categories of CI policies are divided into four levels with different colours. Regulatory policies and standardized policies are the two fundamental ones enforced by the government as the critical and indispensable cornerstone for the CI promotion to the construction industry [32,91]. These two policies reflect the highest degree of mandatory governance in the CI area, which have a strong implementation effect and can fundamentally affect the construction area. They serve as the foundations of CI development and provide a basis for upper knowledge of policies, which provide guidance to all stakeholders and clarify the industrial standards. Upon these, at the middle level, the application scope of CI is further expanded through the promotional policies and three implementation practices, namely urban design and planning policies, technological policies, managerial and educational policies. Remarkably, promotional policies have been introduced into the CI development process as they play a crucial role in connecting the middle level and upper level of sustainability policies. Only with the presence of promotional incentives, the flourish of CI could be pursued comprehensively, such as fresh technological innovations, rational urban planning, and effective management and educational mechanisms. Sustainability policies at the top level represent the highest pursuit of construction worldwide.

Secondly, the seven categories are connected with solid lines and dotted lines accordingly, to illustrate the information extracted from 105 CI-related papers. Solid lines represent direct relationships. Namely, regulatory policies focus on the restrictions and regulations on the CI implementation, which inevitably involves the standardized policies which define the standards in CI. On the one hand, many regulatory policies mention the CI with urban design systems and design strategies as CI can be seen as a solution to fit future urban areas. The regulatory policies, on the other hand, provide the basis for managerial and education policies. For instance, mandatory regulations and intervention strategies direct the risk management and performance management policies among enterprises. Furthermore, managerial and educational policies could lead to the progress of sustainable industrialization. An important part of the lower level of standardized policies is to formulate the technical operations and enhance the technology and innovations of CI. Meanwhile, better-standardized policies can promote industrialization in future urbanization. Technological policies such as efficient government supervision systems and technological innovations can help the pursuit of sustainability policies. Dotted lines represent the potential relationships of policies. For instance, promotional policies play an essential role in stimulating M&EP, UrP, TechP and SusP, as they are developed by governments worldwide to guide and assist markets and serve as the mainstay for other policies.

Overall, the successful implementation of higher-level policies will vitalize the further maturing of lower-level policies in virtuous cycles and vice versa. CI policies are categorized by their features, but they accelerate the CI development cooperatively in essence. Shifting from traditional construction to sustainable construction has gained increasing attention in both academia and industry [50,126]. Practitioners and governments have been looking for sustainable ways to design, build, construct, and operate in the AEC industry. No matter which construction methods are adopted, the ultimate goal should be obtaining sustainable, green, and robust development. Therefore, sustainability policies have been given the sustainable blossoming of humanity. The development tendency of CI policy should unswervingly adhere to sustainable development and rely on regulatory policies and standardized policies as the essential cornerstone.

## 5. Conclusions

Construction industrialisation is an innovative and sustainable mode of construction. By adopting the concept of ‘factory assembly followed by onsite installation,’ CI helps alleviate some of the challenges currently faced by the construction industry. The surge of CI has ameliorated the status of the construction sector in housing crises and lagging productivity. Given the significance of CI in improving construction quality and efficiency, and strong environmental performance, many countries and regions are promoting CI along with relevant policies and regulations. Previously studies primarily focused on exploring the barriers or critical success factors for the CI implementation while ignoring the in-depth and detailed summary of the CI policy’s tendency and the interrelationship of policies from an overall perspective. The need for comprehensive CI policies, including the sustainable development debate over different contexts, are now at the centre of social and economic dialogue. This paper provides a comprehensive literature review of 105 published articles that have mentioned CI policies during the past three decades and sheds light on policy essentials.

Since CI policies are jurisdiction-specific, the background of CI policies in some regions (HK, Mainland China, Japan, Malaysia, and Singapore) was broadly depicted. The annual publications trend in CIP-related research highlights that this area has gained a rising interest among researchers over the last decade. Six developing and developed economies include Mainland China, EU, Australia, the USA, Malaysia, and HK, are found to be the leading contributors to the development of CIP research, with their substantial proportion of the CIP-related studies. It is worth mentioning that China is the dominant contributor to the CI research due to the fact that the current situation of China’s construction market is in the stage of rapid development of CI. Regarding the citation analysis, the three most influential academic articles by Mao et al. [5,47] and Chiang et al. [10] are attributed to the most productive research institutions in this domain, Chongqing University and the Hong Kong Polytechnic University.

Through a detailed and comprehensive exploration analysis and repetitive assessment, seven themes of formulation orientation in CI policies which were concluded from relevant studies are identified, as follows: (1) regulatory policies; (2) standardized policies; (3) promotional policies; (4) urban design and planning policies; (5) technological policies; (6) managerial and educational policies; and (7) sustainability policies. Among these policies, regulatory policies and standardized policies are the two indispensable cornerstones; utilizing promotional policies, in the middle level, urban design and planning policies, technological policies, managerial and educational policies, as implementation approaches, further expands the CI application; sustainability policies should be the most important and have the highest level for the sustainable development of humanity. The interrelation and mutual influence among the seven policies become unambiguous.

Nowadays, the maturing of the CI has profoundly changed the mindset of the incumbents, radically reshaped the way people build, and is currently experiencing a new wave of attention and investment. This study provides an overview of the CI policies for research development and explores the interplay and confluence of seven categories, which provides governments and CI practitioners with a comprehensive understanding of the impact of different policies on CI implementation and inspires decision-makers to examine the realization of policy targets. The interrelationship of categorized policies also assists scholars in enhancing their grasp of current CI policy research and guiding future research on CI. On the other hand, this state-of-the-art literature review helps spur on policy, finance and action, supporting construction industrialisation, which will further have a significant and positive impact on the whole construction industry. The promise of truly hard-headed and sustainable construction industrialisation is well within reach.

However, it is necessary to note some limitations of this study. The selected academic articles provide a snapshot of the current stage of CI policies and advance future research. As time goes on, more themes may spring up along with the public’s updated concerns, which will lead to a continuity of this study. Additionally, issues and disputes that occurred throughout the CI practice have not been elaborated, as this study aims to investigate the documentation and enlightenment from the existing publications. Despite these limitations, this study provides valuable direction and reveals how governments shall give full play to fostering success in CI, leading to a prosperous and sustainable future for the construction industry.

## Figures and Tables

**Table 1:** Most frequently cited journals on CIP.

Journal	Total Times	Number of Papers	Times per Paper
Journal of Cleaner Production	534	18	29.67
Energy and Buildings	219	3	73.00
Habitat International	193	2	96.50
Journal of Management in Engineering	184	2	92.00
Resources Conservation and Recycling	110	4	27.50
Journal of Construction Engineering and Management	81	5	16.20
Engineering Construction and Architectural Management	77	3	25.67
Sustainability	70	10	7.00
Journal of Construction Engineering and Management ASCE	69	2	34.50
Sustainable Cities and Society	61	4	15.25
Land Use Policy	40	3	13.33

The counts ended in July 2021.

**Table 2:** Most frequently cited papers on CIP.

Document Title	Total Citations	Mean Citations per Year
Major Barriers to Offsite Construction: The Developer’s Perspective in China [5].	151	21.57
Comparative study of greenhouse gas emissions between offsite prefabrication and conventional construction methods: Two case studies of residential projects [47].	186	20.67
A holistic review of offsite construction literature published between 2008 and 2018 [50].	79	19.75
A SWOT analysis for promoting offsite construction under the backdrop of China’s new urbanisation [51].	64	16.00
Barriers to the transition towards offsite construction in China: An Interpretive structural modeling approach [52].	60	15.00
A comparative study of environmental performance between prefabricated and traditional residential buildings in China [48].	104	14.86
Evaluating the transition towards cleaner production in the construction and demolition sector of China: A review [53].	52	13.00
Barriers to the adoption of modular integrated construction: Systematic review and meta-analysis, integrated conceptual framework, and strategies [14].	23	11.50
Exploring the challenges to industrialized residential building in China [49].	80	10.00
Measuring the impact of prefabrication on construction waste reduction: An empirical study in China [54].	77	9.63
Implementing onsite construction waste recycling in Hong Kong: Barriers and facilitators [55].	19	9.50
Comparative analysis of modular construction practices in mainland China, Hong Kong and Singapore [56].	19	9.50
Adoption of prefabricated housing-the role of country context [57].	50	8.33
BIM Investment, Returns, and Risks in China’s AEC Industries [58].	41	8.20
Searching for an optimal level of prefabrication in construction: An analytical framework [59].	30	7.50

The counts ended in July 2021.

## Data Availability

Data are contained within the article.
